# Methodological, reporting, and evidence quality of systematic reviews of traditional Chinese medicine for ischemic stroke

**DOI:** 10.3389/fphar.2023.1047650

**Published:** 2023-02-09

**Authors:** Shouyuan Sun, Liang Zhao, Xiaoli Zhou, Xuewu Liu, Zongzhi Xie, Jun Ren, Baoyuan Zhou, Yawen Pan

**Affiliations:** ^1^ Lanzhou University Second Hospital, Lanzhou, China; ^2^ School of Nursing, Lanzhou University, Lanzhou, China; ^3^ No.1 Hospital of Longnan City, Longnan, China; ^4^ The First People’s Hospital of Baiyin, Baiyin, China

**Keywords:** ischemic stroke, traditional Chinese medicine, systematic reviews, meta-analysis, quality

## Abstract

**Objective:** The aim of this study is to critically appraise whether published systematic reviews/meta-analyses of traditional Chinese medicine for adults with ischemic stroke are of sufficient quality and to rate the quality of evidence using the Grading of Recommendations, Assessment, Development, and Evaluation approach.

**Method:** A literature search was performed in the Cochrane Library, PubMed, Chinese National Knowledge Infrastructure, and SinoMed databases by March 2022. The inclusion criteria were systematic reviews/meta-analyses of traditional Chinese medicine in adults who suffered from ischemic stroke. A Measurement Tool to Access Systematic Reviews 2 (AMSTAR-2) and Preferred Reporting Items for Systematic Reviews and Meta-Analyses for Abstract (PRISMA-A) statements were used to assess the methodological and reporting quality of the included reviews. The Grading of Recommendations, Assessment, Development, and Evaluation system was utilized to assess each report’s evidence level.

**Results:** Of the 1,908 titles and abstracts, 83 reviews met the inclusion criteria. These studies were published between 2005 and 2022. The results of AMSTAR-2 showed that 51.4% of the items were reported, but the registration, reasons for the inclusion of study design, the list of excluded studies, and funding information were ignored in the majority of the reviews. The results of PRISMA-A showed that 33.9% of items were reported, and the information on registration, limitation, and funding was not available in many publications. The assessment of the evidence with the Grading of Recommendations, Assessment, Development, and Evaluation showed that more than half (52/83) of the included studies had either low or very low levels of evidence.

**Conclusion:** The reporting quality in the abstract of systematic reviews/meta-analyses on traditional Chinese medicine for ischemic stroke is poor and does not facilitate timely access to valid information for clinical practitioners. Although the methodological quality is of a medium level, this evidence lacks certainty, especially with a high risk of bias in individual studies.

## 1 Introduction

Stroke, a medical disorder in which poor blood flow to the brain leads to cell death, can result in lasting brain injury, long-term disability, and even death. It is the second leading cause of death and the third leading cause of disability-adjusted life-years (DALYs) lost in the world (Feigin et al., 2022). There are two types of stroke, namely, ischemic stroke (due to insufficient blood flow) and hemorrhagic stroke (due to bleeding). Approximately 87% of all strokes are ischemic strokes, where blood flow to the brain is blocked (Tsao et al., 2022). Globally, there are over 77 million prevalent ischemic strokes, 7.6 million new cases, 3.3 million deaths from ischemic stroke, and over 63 million DALYs due to ischemic stroke as of 2019 (GBD 2019 Stroke Collaborators, 2021). The financial burden of ischemic stroke on health services and societies is enormous, and the average lifetime cost is $67,900 (Strilciuc et al., 2021). Reperfusion therapy is considered the standard management and is globally approved for ischemic stroke; however, it is urgent and high-risk (Campbell et al., 2019). Although many drugs have proven to be neuroprotective in preclinical studies, most of them have failed in clinical trials (Gaire, 2018; Campbell et al., 2019).

In recent years, several systematic reviews have been published on the efficacy and safety of traditional Chinese medicine (TCM). It has been suggested that TCM may improve cerebral microcirculation in the brain (Gong and Sucher, 1999), protect against ischemic reperfusion injury (Lee et al., 2005; Wang et al., 2005), reduce oxidative stress reaction (Bi et al., 2012), possess neuroprotective properties, and inhibit apoptosis (Kim, 2005).

High-quality systematic reviews/meta-analyses can provide a more valuable reference for clinical practices than individual studies (Schalken and Rietbergen, 2017; Vrieze, 2018); however, some studies have indicated that the reporting and methodological quality of systematic reviews on TCM are low, which may disturb and even mislead clinical practices or scientific research (Ma et al., 2011; Wang Y. et al., 2016; Zhou et al., 2016). Therefore, the present review aims to evaluate the methodological and reporting quality of systematic reviews/meta-analyses of randomized controlled trials (RCTs) on TCM for ischemic stroke with PRISMA-A (Page et al., 2021) and AMSTAR-2 (Shea et al., 2017). Additionally, the Grading of Recommendations, Assessment, Development, and Evaluation (GRADE) system was used to rate each report’s evidence level to provide evidence to practitioners and researchers when they make clinical decisions.

## 2 Materials and methods

### 2.1 Literature search and selection

**FIGURE 1:**
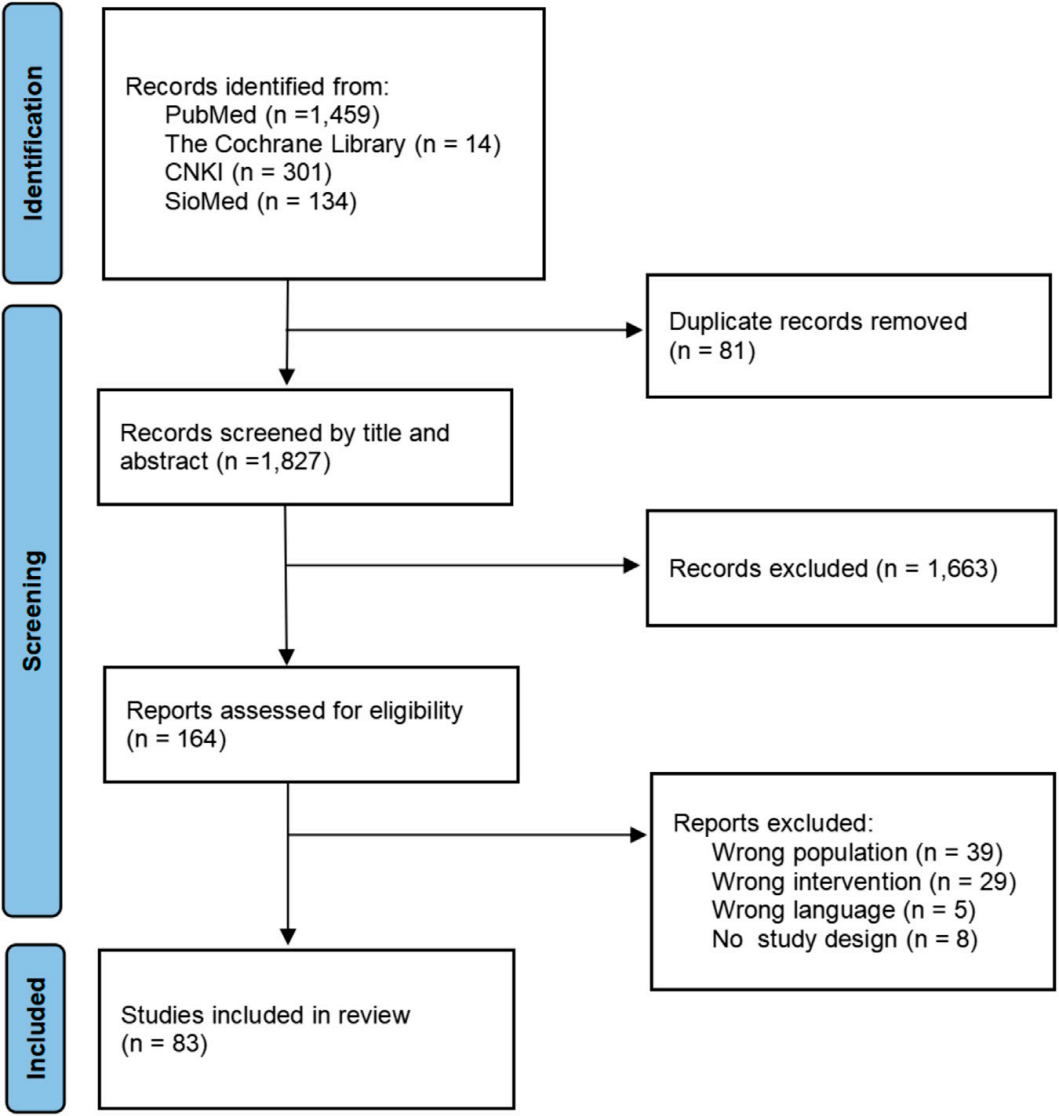
Process of literature selection.

A literature search was performed in PubMed and the Cochrane Library using the keywords “ischemic stroke,” “Chinese medicine,” “systematic review,” and “meta-analysis.” Considering the interventions of Chinese medicine, we conducted additional searches in the Chinese National Knowledge Infrastructure (CNKI) and China Biomedical Literature Service System (SinoMed) with the same keywords (the search strategy is shown in Table 1). We also scanned the reference list mentioned in the related reviews to ensure that our search did not miss any important potential studies on the Chinese medical treatment for ischemic stroke. The deadline for database searching was 1 March 2022. Two steps were adopted for selection. First, the titles and abstracts of the literature were scanned to screen all related studies, and this process was conducted with an online tool known as Rayyan (https://rayyan.ai/), a web and mobile app that was developed specifically to expedite the initial screening of abstracts and titles using a process of semi-automation (Ouzzani et al., 2016). Before the formal screening, the first three authors conducted two rounds of pilot trials for selection with 100 records until the consistency was over 90%. In the formal stage, the first two authors screened all the titles and abstracts independently with the blind function on. When they completed all the records, the blind function turned on, and any disagreements were solved by a discussion with the third author. Then, they located and screened the full texts to identify the reports which met all inclusion criteria in Excel software. The results of the literature search and the selection of articles based on the PRISMA flow diagram are shown in Figure 1.

**TABLE 1:** Search strategy of each database.

Database	Search strategy	Record
PubMed	#1 {[‘‘Systematic Review’’ (Publication Type)] OR [‘‘Systematic Review’ ’(Title/Abstract)]}	1,459
	#2 {[‘‘Ischemic Stroke’’ (MeSH Terms)] OR [‘‘Ischemic Stroke’’ (Title/Abstract)]}	
	#3 #1 AND #2	
Cochrane	‘‘Ischemic stroke’’ in Title Abstract Keyword AND ‘‘Systematic Review’’ in Title Abstract Keyword	14
CNKI	(主题 = 缺血性脑卒中) AND (主题 = 系统评价)	301
SinoMed	‘‘缺血性脑卒中’’ [常用字段:智能] AND ‘‘系统评价’’ [常用字段:智能]	134

To be included in our review, a review had to meet the following criteria: 1) the patients included were adults who experienced ischemic stroke. The transient ischemic attack and hemorrhagic stroke were also excluded; 2) the interventions included were the Chinese medical treatment, where acupuncture and massage were excluded. Single TCM treatments, integrated TCM treatments, and integrated TCM and Western medicine treatments were included, and animal experiments were excluded; 3) the comparisons were of TCM, Western medicine treatment, placebo, or no treatment; 4) the outcome without limitations; 5) the study design included was the systematic review or meta-analysis. The overview of systematic reviews, mixed-method review, qualitative review, integrative review, rapid review, and evidence synthesis were beyond the inclusion; 6) published in English and Chinese; and 7) there was no restriction on the time framework of the studies.

### 2.2 Information extraction and coding

The information extraction and coding were designed with Excel software, and two rounds of pilots were carried out before the formal extraction and coding to make sure the extractors have an accurate and consistent understanding of what to extract. When the consistency reaches 100%, the formal data extraction and coding process begins. There were four parts of information: (Feigin et al., 2022) general information, including title, author, language, the year of publication, database, number of included studies, number of patients, type of interventions and comparisons, and outcomes; (Tsao et al., 2022) information of 12 items in PRISMA-A; (GBD 2019 Stroke Collaborators, 2021) information of 16 items in AMSTAR-2; and (Strilciuc et al., 2021) information of five domains in GRADE. The PRISMA-A and AMSTAR-2 were coded into “Yes,” “Partial Yes,” or “No” depending on how well each item was reported. The fourth part was rated as “Not serious,” “Serious,” “Very serious,” or “No information,” according to the risk bias in each domain. “High,” “Moderate,” “Low,” and “Very Low” were applied to rate the overall level of risks of bias. The process of data extraction and coding was performed by two authors independently, and any disagreements were solved by the third author.

### 2.3 Data analysis

We used the PRISMA-A guideline to reflect the abstract quality of systematic reviews/meta-analyses, and descriptive statistics (frequencies and percentages) was used to describe the reporting characteristics. We adopted AMSTAR-2 to evaluate the methodological quality of reviews, and the results were shown with percentages for each item. We also applied GRADE to rate the overall quality of evidence, and the ratio of each grade was analyzed (high, moderate, low, and critically low). In addition, regression analysis with the year of publication and the percentage of “Yes” in PRISMA-A and AMSTAR-2 was performed to explore the changes in quality, and subgroup analysis was adopted to compare the difference depending on the publication language. All these analyses were conducted in Excel software and SPSS 22.0.

## 3 Results

### 3.1 Characteristics of included studies

Of 1,908 records, 1,827 titles and abstracts were screened with Rayyan after removing the duplications, 52 studies with disagreements were resolved by the third author (the consistency is 97.2%), 164 were located with full text, and 83 systematic reviews/meta-analyses on the TCM for ischemic stroke were included which were published between 2005 and 2022. The PRISMA flow diagram is shown in Figure 1. Among them, 61 (73.5%) were published in Chinese, 81 (97.6%) were conducted in China, 1 (1.2%) in Malaysia, and 1 (1.2%) in Korea. The number of included primary studies in these reviews ranged from two to 107. A total of 43 (51.8%) were focused on acute ischemic stroke (AIS). The number of patients involved in the reports ranged from 161 to 13,073. The characteristics of included studies are shown in Table 2.

**TABLE 2:** Characteristics of included studies.

Study ID	Country	Publication language	Patients	Number of included studies	Sample	Intervention	Comparison	Outcome
Ma, 2005	China	Chinese	AIS	54	4,421	TCM	NT and placebo	Clinically effective rate, hemorheology, and blood lipid level
Li, 2006	China	Chinese	AIS	25	2,571	TCM	NT	Clinically effective rate and neurological deficit
Wang, 2006	China	Chinese	AIS	11	1,496	TCM	TCM and TCM + WM	Mortality and clinically effective rate
Chen, 2007	China	Chinese	AIS	14	1,119	TCM + WM	CM	Disability and neurological deficit
Li, 2007	China	Chinese	AIS	4	409	TCM	TCM	Degree of neurological deficit
Yuan, 2008	China	English	AIS	2	161	TCM	TCM and NT	Degree of neurological deficit
Lei, 2010	China	Chinese	AIS	13	1,296	TCM	TCM and NT	Clinically effective rate, neurological deficit, hemorheology
Peng, 2010	China	Chinese	AIS	29	3,191	TCM	TCM	Quality of life, neurological deficit, hemorheology, and adverse reactions
Ding, 2011	China	Chinese	AIS	10	938	TCM	TCM and placebo	Clinically effective rate and neurological deficit
Liu, 2011	China	Chinese	AIS	NR	1,280	TCM	TCM	Clinically effective rate, neurological deficit, and adverse reactions
Cheng, 2012	China	English	AIS	7	545	TCM	TCM	Neurological deficit, mortality, neurological deficit, and cytokine level
Zhao, 2012	China	Chinese	AIS	21	2,014	TCM	NT and placebo	Neurological deficit and clinically effective rate
Fu, 2013	China	English	AIS	8	601	TCM	WM	Neurological deficit and clinically effective rate
Han, 2013	China	English	AIS	28	2,385	TCM + WM	WM	Clinically effective rate, activities of daily living, neurological deficit, TCM symptoms, and hemorheology
Ma, 2013	China	Chinese	AIS	14	1,218	TCM	TCM	Clinically effective rate and neurological deficit
Zhou, 2013	China	Chinese	AIS	39	3,906	TCM	TCM	Mortality, dependence, neurological deficit, and adverse reactions
Fan, 2014	China	English	AIS	7	762	TCM	TCM	Degree of neurological deficit
Ma, 2014	China	Chinese	AIS	10	720	TCM	TCM	Clinically effective rate and neurological deficit
Wan, 2015	China	Chinese	AIS	9	631	TCM	TCM	Clinically effective rate and neurological deficit
Liu, 2016	China	English	AIS	12	970	TCM	TCM	Activities of daily living, neurological deficit, and clinically effective rate
Qi, 2016	China	Chinese	AIS	24	3,060	TCM + WM	WM	Neurological deficit and hemorheology
Zheng, 2016	China	Chinese	AIS	26	2,831	TCM + WM	WM	Clinically effective rate, neurological deficit, activities of daily living, protein level, hemorheology, TCM symptoms
Zhang, 2017	China	Chinese	AIS	6	451	TCM	TCM	Clinically effective rate and neurological deficit
Zhang, 2017	China	Chinese	AIS	36	2,821	TCM + WM	WM	Neurological deficit, hemorheology, blood lipid level, protein level and clinically effective rate
Zhu, 2017	China	Chinese	AIS	20	2,237	TCM	TCM and WM + TCM	Neurological deficit and clinically effective rate
Han, 2018	Korea	English	AIS	80	8,057	TCM	TCM	Neurological deficit, sports function, and activities of daily living
Chen, 2018	China	Chinese	AIS	10	981	TCM	TCM	Clinically effective rate, neurological deficit, and hemorheology
Wang, 2018	China	Chinese	AIS	16	1,687	TCM	TCM	Neurological deficit, Clinically effective rate
Zhao, 2018	China	Chinese	AIS	13	1,330	TCM	TCM	Clinically effective rate and neurological deficit
Cai, 2019	China	Chinese	AIS	9	959	TCM + WM	WM	Clinically effective rate, neurological deficit, activities of daily living, and hemorheology
Chang, 2019	China	Chinese	AIS	15	1,619	TCM + WM	WM	Neurological deficit and activities of daily living
Huang, 2019	China	Chinese	AIS	24	2,154	TCM	TCM + WM	Clinically effective rate, neurological deficit, hemorheology, and protein level
Chong, 2020	Malaysia	English	AIS	12	1,466	TCM	TCM	Mortality, activities of daily living, neurological deficit, and adverse reactions
Liu, 2020	China	Chinese	AIS	26	1,994	TCM	WM	Neurological deficit, TCM symptoms, and clinically effective rate
Lv, 2020	China	Chinese	AIS	35	4,379	TCM	TCM and TCM + WM	Degree of neurological deficit, activities of daily living, and hemorheology
Yao, 2020	China	Chinese	AIS	21	1,722	TCM	TCM	Clinically effective rate, activities of daily living, neurological deficit, TCM symptoms, hemorheology, and protein level
Wang, 2021	China	English	AIS	43	4,170	TCM + WM	WM	Degree of neurological deficit, activities of daily living, overall response rate, hemorheology, coagulation function, and adverse reactions
Cheng, 2021	China	Chinese	AIS	19	1,768	TCM + WM	TCM	Clinically effective rate, neurological deficit, cognitive ability, adverse reactions, coagulation function, and hemorheology
Huang, 2021	China	Chinese	AIS	17	1,327	TCM + WM	WM	Clinically effective rate, neurological deficit, and activities of daily living
Luo, 2021	China	Chinese	AIS	9	765	TCM + WM	TCM + WM	Neurological deficit, clinically effective rate, quality of life, activities of daily living, and adverse reactions
Meng, 2021	China	Chinese	AIS	20	2,059	TCM + WM	WM	Clinically effective rate, neurological deficit, activities of daily living, and adverse reactions
Tang, 2021	China	Chinese	AIS	22	2,078	TCM + WM	WM	Clinically effective rate, motor function, protein level, hemorheology, and adverse reactions
Zhou, 2022	China	English	AIS	17	1,489	TCM	TCM	Clinically effective rate, neurological deficit, activities of daily living, and hemorheology
Wu, 2005	China	Chinese	IS	3	304	TCM	TCM	Degree of neurological deficit
Li, 2007	China	Chinese	IS	22	2,488	TCM	TCM, WM + TCM, and placebo	Clinically effective rate, neurological deficit, and hemorheology
Yang, 2008	China	Chinese	IS	46	4,808	TCM and TCM + WM	TCM	Clinically effective rate, adverse reactions, and quality of life
Hao, 2012	China	English	IS	19	1,580	TCM + WM	WM	Clinically effective rate and neurological deficit
Ni, 2013	China	English	IS	3	643	TCM	TCM	Recurrence rate
Ni, 2013	China	Chinese	IS	7	679	TCM	TCM	Clinically effective rate, neurological deficit, and hemorheology
Lu, 2014	China	English	IS	12	728	TCM	WM	Clinically effective rate and neurological deficit
Liu, 2014	China	Chinese	IS	9	925	TCM	TCM	Neurological deficit
Yang, 2015	China	English	IS	9	931	TCM	TCM	Mortality, recurrence rate, and adverse reactions
Zeng, 2015	China	Chinese	IS	6	1,574	TCM	TCM	Clinically effective rate, neurological deficit, protein level, and cytokine level
Xie, 2015	China	Chinese	IS	22	1,976	TCM + WM	WM, TCM	Recurrence rate, activities of daily living, neurological deficit, and clinically effective rate
Xie, 2015	China	Chinese	IS	28	2,854	TCM + WM and TCM	TCM and TCM + WM	Activities of daily living, neurological deficit, motor function, and clinically effective rate
Zhou, 2015	China	Chinese	IS	27	2,908	TCM, TCM + TCM, and TCM + WM	WM, TCM, WM + TCM, and placebo	Clinically effective rate, neurological deficit, and activities of daily living
Zeng, 2016	China	Chinese	IS	19	2,383	TCM	TCM	Clinically effective rate, neurological deficit, activities of daily living, and protein level
Hou, 2016	China	Chinese	IS	9	620	TCM + WM	WM	Clinically effective rate and neurological deficit
Li, 2016	China	Chinese	IS	18	4,416	TCM	TCM and TCM + placebo	Recurrence rate, clinical effective rate, mortality, and adverse reactions
Feng, 2017	China	Chinese	IS	17	1,550	TCM	NT	Activities of daily living and neurological deficit
Xiang, 2017	China	Chinese	IS	8	822	TCM + WM	WM	Clinically effective rate, neurological deficit, and hemorheology
Yang, 2018	China	English	IS	14	5,206	TCM + WM	WM	Adverse reactions
Ding, 2018	China	Chinese	IS	75	6,904	TCM	TCM	Clinically effective rate, neurological deficit, TCM symptoms, activities of daily living, and adverse reactions
Wang, 2018	China	Chinese	IS	107	13,073	TCM	TCM	Clinically effective rate, TCM symptoms, neurological deficit, activities of daily living, mortality, recurrence rate, and adverse reactions
Xu, 2018	China	Chinese	IS	39	3,539	TCM and TCM + WM	TCM and TCM	Neurological deficit, motor function, activities of daily living, hemorheology, protein level, cytokine level, and TCM symptoms
Xue, 2019	China	English	IS	39	3,182	TCM + WM	WM	Overall response rate, neurological deficit, protein level, hemorheology, blood lipid level, and adverse reactions
Zhang, 2019	China	English	IS	13	1,275	TCM + WM	WM	Clinically effective rate, neurological deficit, blood lipid level, and atherosclerotic plaque area
Liu, 2019	China	Chinese	IS	3	3,541	TCM	WM	Neurological deficit and clinically effective rate
Wang, 2019	China	Chinese	IS	18	1,570	TCM	TCM	Clinically effective rate, neurological deficit, and hemorheology
Yu, 2019	China	Chinese	IS	9	1,511	TCM	WM	Clinically effective rate, neurological deficit, activities of daily living, and protein level
Ji, 2020	China	English	IS	15	1,829	TCM	TCM	Dependence and neurological deficit
Zhong, 2020	China	English	IS	40	3,260	TCM + WM	WM	Neurological deficit, activities of daily living, protein level, hemorheology, blood lipid level, clinical effective rate, and adverse reactions
Li, 2020	China	Chinese	IS	13	1,274	TCM + WM	WM	Clinically effective rate, neurological deficit, and inflammation factor
Zhou, 2020	China	Chinese	IS	29	3,682	TCM + WM	WM and TCM + WM	Clinically effective rate, neurological deficit, and hemorheology
Ling, 2021	China	English	IS	11	1,644	TCM + WM	WM	Clinically effective rate, neurological deficit, hemorheology, and adverse reactions
Li, 2021	China	English	IS	11	1 084	TCM	TCM and WM	Neurological deficit and activities of daily living
Guan, 2021	China	Chinese	IS	16	824	TCM + WM	WM	Neurological deficit, cognitive ability, activities of daily living, and clinically effective rate
Ding, 2021	China	Chinese	IS	16	1,616	TCM	TCM and sham-TCM	Clinically effective rate, neurological deficit, TCM symptoms, sports function, and activities of daily living
Dong, 2021	China	Chinese	IS	27	2,668	TCM	TCM	Clinically effective rate, neurological deficit, activities of daily living, adverse reactions, and protein level
Guan, 2021	China	Chinese	IS	16	1,615	TCM + WM	WM	Neurological deficit, cognitive ability, activities of daily living, and clinically effective rate
Liao, 2021	China	Chinese	IS	18	7,951	TCM	TCM	Recurrence rate, mortality, neurological deficit, activities of daily living, and disability, protein level
Liu, 2022	China	English	IS	28	6,683	TCM + WM	WM and placebo + WM	Neurological deficit, mortality, recurrence rate, adverse reactions, activities of daily living, clinically effective rate, cognitive ability, coagulation function, hemorheology, blood lipid level, and quality of life
Guo, 2022	China	Chinese	IS	9	1,005	TCM + WM	WM	Clinically effective rate, neurological deficit, cognitive ability, atherosclerotic plaque area, cerebral blood flow velocity, activities of daily living, cytokine level, and adverse reactions

IS: ischemic stroke; AIS: acute ischemic stroke; NT: no treatment*;* CM: conventional treatment; TCM: traditional Chinese medicine; and WM: Western medicine.

### 3.2 Reporting quality of abstracts

**FIGURE 2:**
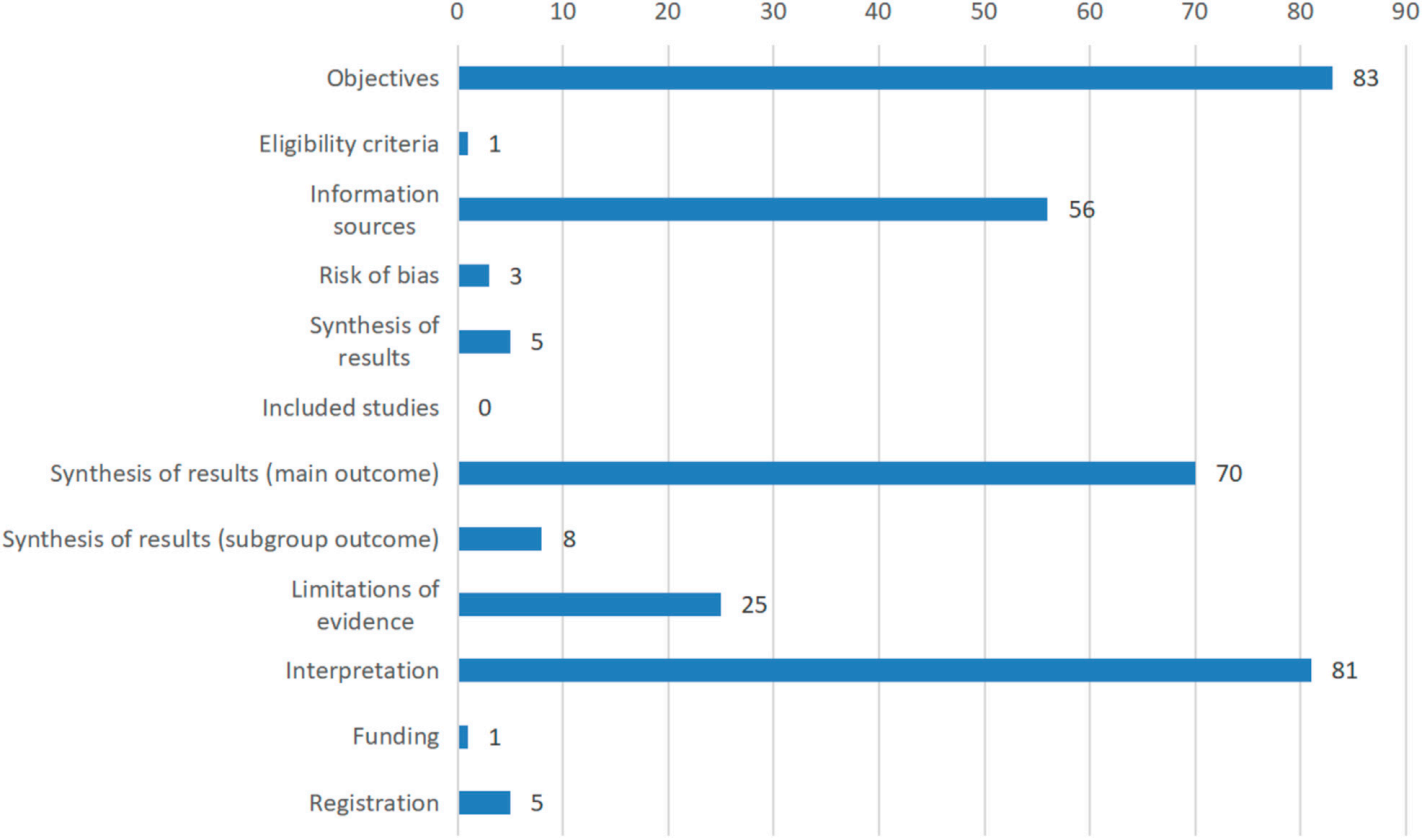
Reporting quality of abstracts based on PRISMA-A.

Based on the PRISMA-A statement, two reviewers discussed the different coding items with the third author, and finally unified the coding data. The results showed that among 83 abstracts that were accessed, the overall total report (percentage of “Yes”) was 33.9% and the partial report (percentage of “Partial Yes”) was 13.2%. Most reviews (96.6%) clearly described the publication type and all of them reported the objectives of the reviews, while only information sources were reported by over half reviews in the methods section. All reviews demonstrated the results (84.3% in the main outcome) and discussion (97.6% in interpretation), but the limitations of evidence were ignored by many publications (30.1% reported). In addition, other information such as funding and registration were mentioned by 1.2% and 6.0%, respectively. The completion of a single item is shown in Figure 2, and the results of each item are shown in the Supplementary material.

### 3.3 Methodological quality

**FIGURE 3:**
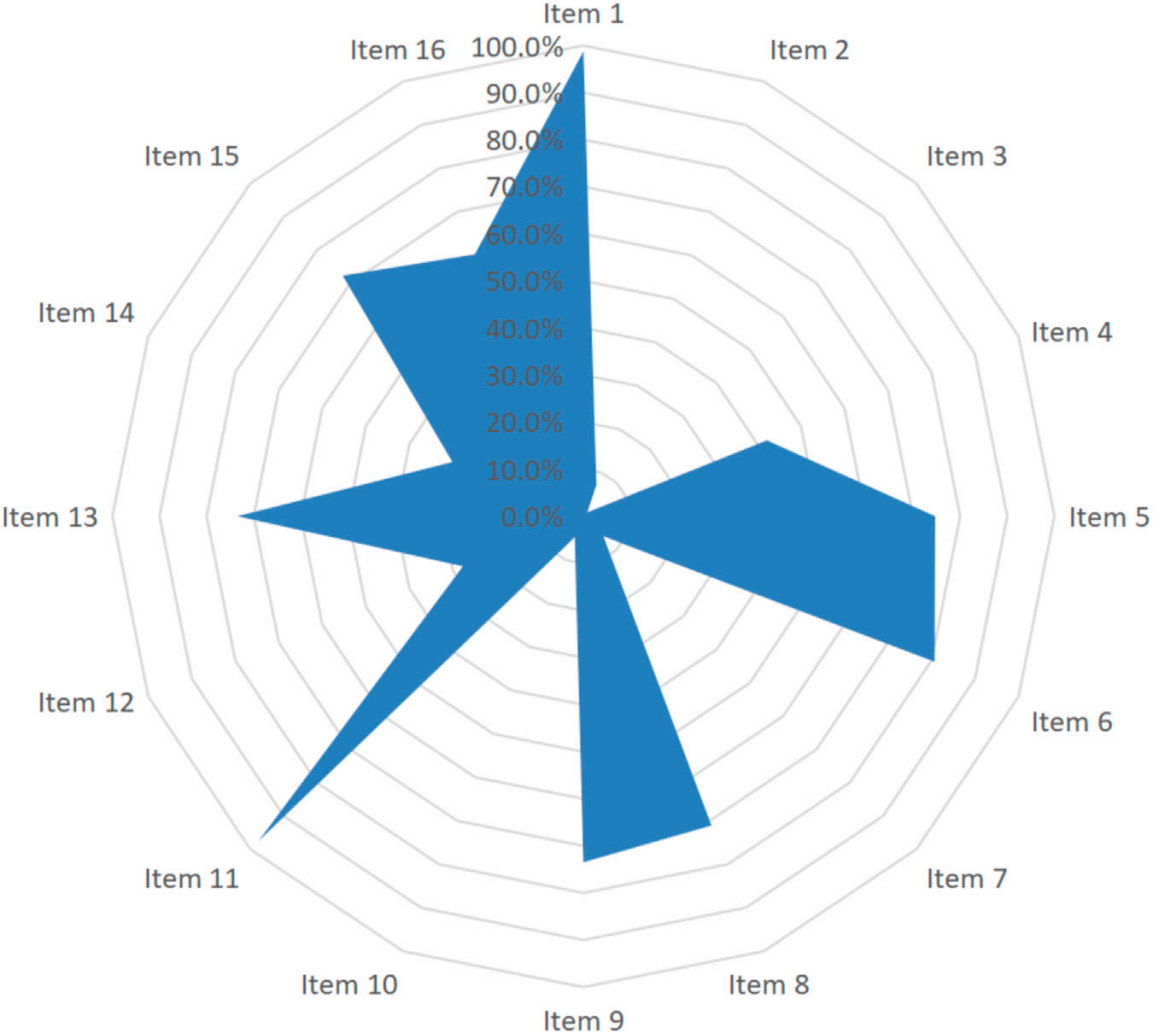
Methodological quality of systematic reviews based on AMSTAR-2.

The methodological quality of reviews was accessed by the AMSTAR-2, including 16 items. The results showed that the average report completeness was 51.4%, and eight items (items 1, 5, 6, 8, 9, 11, 13, and 15) were higher than 70% (71.1%–98.9%). However, fewer papers included in the evaluation fully reported the registration (item 2: 7.2%), the reasonableness of the inclusion criteria of the study design (item 3: 1.2%), the list of excluded studies (item 7: 6.0%), information on the funding (item 10: 4.8%), and the explanation of heterogeneity (item 14: 30.1%), which directly affects the openness and transparency of systematic reviews and results in the inability of follow-up researchers to repeat and update (Figure 3). The results of each item are shown in the Supplementary material.

### 3.4 Evidence certainty

**FIGURE 4:**
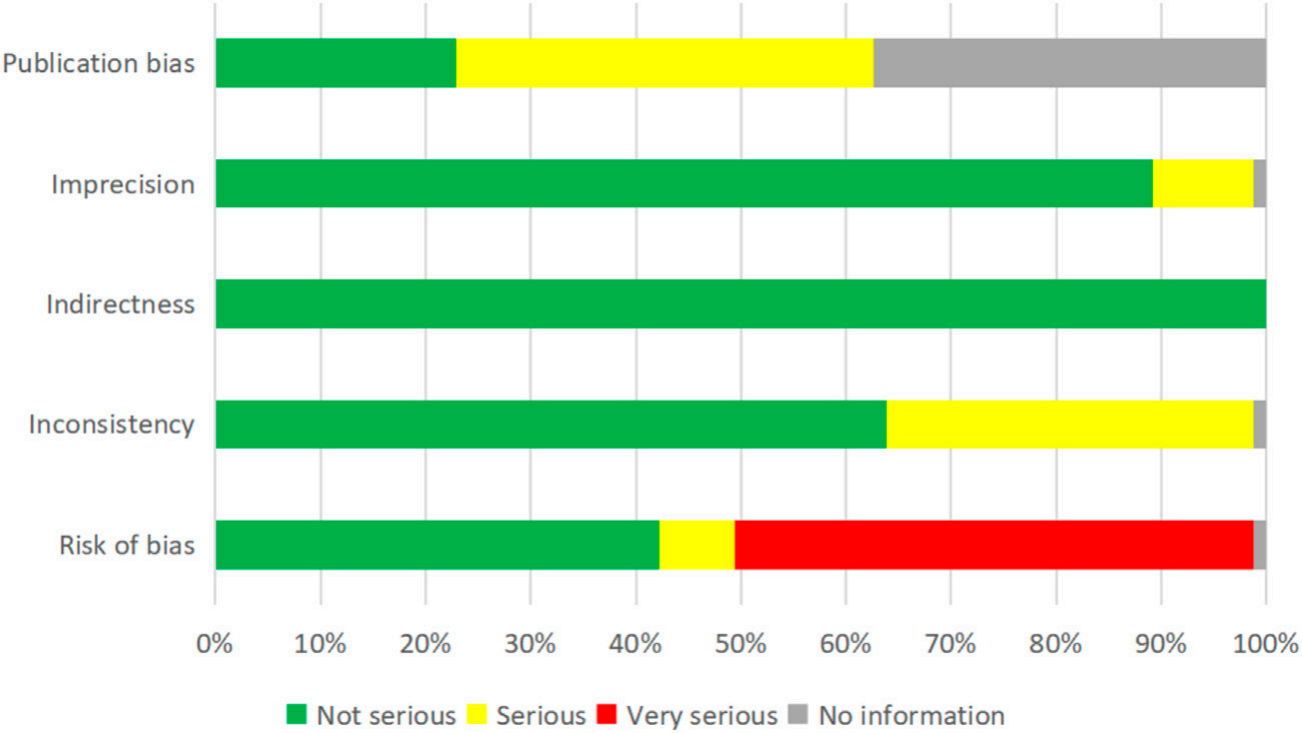
Evidence certainty based on the GRADE system.

We adopted the GRADE system to evaluate the overall evidence quality, and the results showed that there were only 15.7% of included studies in high certainty, 21.7% in moderate, 30.1% in low, and 32.5% in very low certainty. Notably, 49.4% of included reviews had a very serious risk of bias, in which more than one-third of them had serious publication bias and inconsistency, and 9.6% had a serious risk of imprecision (Figure 4). The results of each item are shown in the Supplementary material.

### 3.5 Changes in methodological, reporting, and evidence certainty

#### 3.5.1 Publication year

Table 3 shows that the reporting quality and certainty of meta-analyses on TCM for ischemic stroke had no obvious improvement after PRISMA and AMSTAR-2 were released in 2009, but the methodological quality showed an upward trend, especially in the inclusion/exclusion criteria (item 7, χ2 = 13.3, and *p* < 0.01) and risk of bias assessment (item 12, χ2 = 7.7, and *p* < 0.01).

**TABLE 3:** Results of the subgroup analysis.

	Item	Language			Publication year			Intervention		
		Chinese (%)	English (%)	χ2	≤ 2009 (%)	> 2009 (%)	χ2	TCM (%)	TCM + WM	χ2
PRISMA-A	Item 1						.			
	No	0.0	0.0		0.0	0.0		0.0	0.0%	
	Partial yes	100.0	100.0		100.0	100.0		100.0	100.0%	
	Yes	0.0	0.0		0.0	0.0		0.0	0.0%	
	Item 2			1.7			0.0			0.1
	No	91.8	81.8		88.9	89.2		88.5	90.3%	
	Partial yes	8.2	18.2		11.1	10.8		11.5	9.7%	
	Yes	0.0	0.0		0.0	0.0		0.0	0.0%	
	Item 3			1.1			3.9			0.1
	No	24.6	36.4		55.6	24.3		28.8	25.8%	
	Partial yes	75.4	63.6		44.4	75.7		71.2	74.2%	
	Yes	0.0	0.0		0.0	0.0		0.0	0.0%	
	Item 4			1.5			0.6			1.3
	No	60.7	45.5		44.4	58.1		51.9	64.5%	
	Partial yes	39.3	54.5		55.6	41.9		48.1	35.5%	
	Yes	0.0	0.0		0.0	0.0		0.0	0.0%	
	Item 5			2.5			1.1			0.0
	No	93.4	81.8		100.0	89.2		90.4	90.3%	
	Partial yes	6.6	18.2		0.0	10.8		9.6	9.7%	
	Yes	0.0	0.0		0.0	0.0		0.0	0.0%	
	Item 6			.			.			.
	No	0.0	0.0		0.0	0.0		0.0	0.0%	
	Partial yes	100.0	100.0		100.0	100.0		100.0	100.0%	
	Yes	0.0	0.0		0.0	0.0		0.0	0.0%	
	Item 7			0.1			0.3			0.5
	No	16.4	13.6		22.2	14.9		13.5	19.4%	
	Partial yes	83.6	86.4		77.8	85.1		86.5	80.6%	
	Yes	0.0	0.0		0.0	0.0		0.0	0.0%	
	Item 8			0.0			1.1			0.0
	No	90.2	90.9		100.0	89.2		90.4	90.3%	
	Partial yes	9.8	9.1		0.0	10.8		9.6	9.7%	
	Yes	0.0	0.0		0.0	0.0		0.0	0.0%	
	Item 9			1.7			3.1			1.3
	No	73.8	59.1		44.4	73.0		65.4	77.4%	
	Partial yes	26.2	40.9		55.6	27.0		34.6	22.6%	
	Yes	0.0	0.0		0.0	0.0		0.0	0.0%	
	Item 10			5.7			0.3			1.2
	No	0.0	9.1		0.0	2.7		3.8	0.0%	
	Partial yes	100.0	90.9		100.0	97.3		96.2	100.0%	
	Yes	0.0	0.0		0.0	0.0		0.0	0.0%	
	Item 11			2.8			0.1			0.6
	No	100.0	95.5		100.0	98.6		98.1	100.0%	
	Partial yes	0.0	4.5		0.0	1.4		1.9	0.0%	
	Yes	0.0	0.0		0.0	0.0		0.0	0.0%	
	Item 12			3.1			0.7			0.7
	No	96.7	86.4		100.0	93.2		92.3	96.8%	
	Partial yes	3.3	13.6		0.0	6.8		7.7	3.2%	
	Yes	0.0	0.0		0.0	0.0		0.0	0.0%	
AMSTAR-2	Item 1			2.8			0.1			0.6
	No	0.0	4.5		0.0	1.4		1.9	0.0%	
	Partial yes	0.0	0.0		0.0	0.0		0.0	0.0%	
	Yes	100.0	95.5		100.0	98.6		98.1	100.0%	
	Item 2			17.9**			0.2			0.4
	No	100.0	72.7		88.9	93.2		94.2	90.3%	
	Partial yes	0.0	0.0		0.0	0.0		0.0	0.0%	
	Yes	0.0	27.3		11.1	6.8		5.8	9.7%	
	Item 3			0.4			0.1			0.6
	No	98.4	100.0		100.0	98.6		98.1	100.0%	
	Partial yes	0.0	0.0		0.0	0.0		0.0	0.0%	
	Yes	1.6	0.0		0.0	1.4		1.9	0.0%	
	Item 4			2.6			5.7			5.3
	No	21.3	22.7		33.3	20.3		28.8	9.7%	
	Partial yes	41.0	22.7		0.0	40.5		28.8	48.4%	
	Yes	37.7	54.5		66.7	39.2		42.3	41.9%	
	Item 5			0.1			0.4			6.4*
	No	24.6	27.3		33.3	24.3		34.6	9.7%	
	Partial yes	0.0	0.0		0.0	0.0		0.0	0.0%	
	Yes	75.4	72.7		66.7	75.7		65.4	90.3%	
	Item 6			2.0			0.1			2.9
	No	23.0	9.1		22.2	18.9		25.0	9.7%	
	Partial yes	0.0	0.0		0.0	0.0		0.0	0.0%	
	Yes	77.0	90.9		77.8	81.1		75.0	90.3%	
	Item 7			0.5			13.3**			0.0
	No	95.1	90.9		66.7	97.3		94.2	93.5%	
	Partial yes	0.0	0.0		0.0	0.0		0.0	0.0%	
	Yes	4.9	9.1		33.3	2.7		5.8	6.5%	
	Item 8			5.8			1.8			4.3
	No	3.3	0.0		0.0	2.7		3.8	0.0%	
	Partial yes	32.8	9.1		44.4	24.3		32.7	16.1%	
	Yes	63.9	90.9		55.6	73.0		63.5	83.9%	
	Item 9			4.7			0.4			3.0
	No	1.6	0.0		0.0	1.4		1.9	0.0%	
	Partial yes	31.1	9.1		33.3	24.3		30.8	16.1%	
	Yes	67.2	90.9		66.7	74.3		67.3	83.9%	
	Item 10			1.2			0.9			0.3
	No	96.7	90.9		88.9	95.9		96.2	93.5%	
	Partial yes	0.0	0.0		0.0	0.0		0.0	0.0%	
	Yes	3.3	9.1		11.1	4.1		3.8	6.5%	
	Item 11			0.7			0.3			1.2
	No	3.3	0.0		0.0	2.7		3.8	0.0%	
	Partial yes	0.0	0.0		0.0	0.0		0.0	0.0%	
	Yes	96.7	100.0		100.0	97.3		96.2	100.0%	
	Item 12			0.0			7.7**			0.1
	No	72.1	72.7		33.3	77.0		71.2	74.2%	
	Partial yes	0.0	0.0		0.0	0.0		0.0	0.0%	
	Yes	27.9	27.3		66.7	23.0		28.8	25.8%	
	Item 13			0.0			1.2			2.1
	No	26.2	27.3		11.1	28.4		21.2	35.5%	
	Partial yes	0.0	0.0		0.0	0.0		0.0	0.0%	
	Yes	73.8	72.7		88.9	71.6		78.8	64.5%	
	Item 14			0.0			1.0			0.1
	No	70.5	68.2		55.6	71.6		71.2	67.7%	
	Partial yes	0.0	0.0		0.0	0.0		0.0	0.0%	
	Yes	29.5	31.8		44.4	28.4		28.8	32.3%	
	Item 15			0.3			1.4			0.7
	No	26.2	31.8		44.4	25.7		30.8	22.6%	
	Partial yes	0.0	0.0		0.0	0.0		0.0	0.0%	
	Yes	73.8	68.2		55.6	74.3		69.2	77.4%	
	Item 16			2.0			3.1			8.6**
	No	44.3	27.3		66.7	36.5		51.9	19.4%	
	Partial yes	0.0	0.0		0.0	0.0		0.0	0.0%	
	Yes	55.7	72.7		33.3	63.5		48.1	80.6%	
GRADE	Risk of bias			6.6*			10.3			3.2
	No information	1.6	0.0		0.0	1.4		1.9	0.0%	
	Not serious	49.2	22.7		0.0	47.3		36.5	51.6%	
	Serious	8.2	4.5		0.0	8.1		5.8	9.7%	
	Very serious	41.0	72.7		100.0	43.2		55.8	38.7%	
	Inconsistency			3.4			0.1			6.1*
	No information	0.0	4.5		0.0	1.4		0.0	3.2%	
	Not serious	62.3	68.2		66.7	63.5		73.1	48.4%	
	Serious	37.7	27.3		33.3	35.1		26.9	48.4%	
	Very serious	0.0	0.0		0.0	0.0		0.0	0.0%	
	Indirectness			.			.			.
	No information	0.0	0.0		0.0	0.0		0.0	0.0%	
	Not serious	100.0	100.0		100.0	100.0		100.0	100.0%	
	Serious	0.0	0.0		0.0	0.0		0.0	0.0%	
	Very serious	0.0	0.0		0.0	0.0		0.0	0.0%	
	Imprecision			9.0*			1.9			2.2
	No information	0.0	4.5		0.0	1.4		0.0	3.2%	
	Not serious	95.1	72.7		77.8	90.5		88.5	90.3%	
	Serious	4.9	22.7		22.2	8.1		11.5	6.5%	
	Very serious	0.0	0.0		0.0	0.0		0.0	0.0%	
	Publication bias			2.0			1.6			1.5
	No information	34.4	45.5		55.6	35.1		42.3	29.0%	
	Not serious	21.3	27.3		11.1	24.3		21.2	25.8%	
	Serious	44.3	27.3		33.3	40.5		36.5	45.2%	
	Very serious	0.0	0.0		0.0	0.0		0.0	0.0%	
	Certainty			4.4			6.1			1.9
	Very low	26.2	50.0		55.6	29.7		32.7	32.3%	
	Low	32.8	22.7		44.4	28.4		32.7	25.8%	
	Moderate	23.0	18.2		0.0	24.3		23.1	19.4%	
	High	18.0	9.1		0.0	17.6		11.5	22.6%	

**, *p*< 0.01; *, *p*< 0.05.

#### 3.5.2 Type of intervention

52 (62.7%) reviews included TCM for ischemic stroke, and 29 (34.9%) included TCM with WM. Table 3 shows that the reviews focused on TCM with WM are the most frequently adopted selection in duplicate (item 5 in AMSTAR-2: χ2 = 6.4, and *p* < 0.01) and reported potential sources of conflict of interest (item 16 in AMSTAR-2: χ2 = 8.6, and *p* < 0.01). However, they had a higher risk of inconsistency (χ2 = 6.1 and *p* < 0.01).

#### 3.5.3 Publication language

We included both Chinese (61, 73.5%) and English studies (22, 26.5%) and analyzed the variety of reporting and methodological quality depending on the publication language. The results showed that reviews published in Chinese rarely reported the protocol (item 2 in AMSTAR-2: χ2 = 17.9, and *p* < 0.01). However, studies published in English had a higher risk of bias (χ2 = 6.6 and *p* < 0.05) and imprecision (χ2 = 9.0 and *p* < 0.05) than those with Chinese studies. The details are shown in Table 3.

## 4 Discussion

Due to the advantages of TCM for ischemic stroke, numerous meta-analyses were conducted to evaluate its efficacy and safety. The present review included 83 reviews to evaluate the methodological, reporting, and evidence certainty of systematic reviews and meta-analysis of TCM for ischemic stroke. The results showed that the reporting quality for abstract was in the low level (33.9% of items were reported) and methodological quality was in the middle level (51.4% of items were reported). However, only 15.7% of included studies were in high certainty. In addition, 49.4% of reviews had a very serious risk of bias, 37.1% had serious publication bias, and 34.9% had inconsistency risk, which may affect the evidence confidence and even mislead the clinical decision.

Although the PRISMA and AMSTAR were developed to improve the reporting and methodological quality of systematic review/meta-analysis (Moher et al., 1999; Moher et al., 2009; Shea et al., 2009; Sharma and Oremus, 2018; Page et al., 2021), there was no obvious persistent improvement in the reviews of the TCM for ischemic stroke. Li et al. (2009) introduced the PRISMA in China and translated it to a Chinese vision, but it was interpreted systematically since 2015 (Tian et al., 2015; Zhang et al., 2015; Sun et al., 2018). The majority (item 81: 97.6%) of reviews included in this study were conducted in China, and the delayed promotion of PRISMA in China may be the main reason for this result.

In addition, it was rare that Chinese medicine journals recommended the PRISMA guideline when the authors conducted and submitted their manuscripts to the journal. For example, the *Journal of Traditional Chinese Medicine*, an open-access journal that aims to publish evidence-based, scientifically justified original articles, and review papers in all aspects of Chinese medicine, only suggested that authors should refer to the CONSORT 2010 Statement for reports of RCTs. Even *the American Journal of Chinese Medicine*, an international journal of comparative eastern and western medicine, did not mention the reporting requirement for systematic reviews in the submission guidelines. Therefore, it is necessary for editors to highly recommend the reporting items of systematic reviews in their submission guidelines to promote the repeatability and transparency of Chinese medicine studies.

Differing from Western medicine, most of the principles of TCM were derived from a philosophical basis instead of biological mechanisms, such as the dynamic balance between Yin and Yang, and the majority of treatments aimed to expel or suppress the cause of disharmony and restore balance (Tang et al., 2008). Moreover, the same disease in Western medicine can be categorized into different syndromes (called Zhengs) in TCM. Thus, patients with the same “diagnosis” in Western medicine can be treated differently in TCM, and even the treatments of TCM in the same patient vary over time. Although there were several reporting guidelines for systematic reviews, such as the CONSORT and PRISMA guidelines, the uniqueness of TCM was not considered in the present guidelines (Wang G. et al., 2016; Hu et al., 2019; Yu et al., 2019). For example, in addition to patients, interventions, comparisons, and outcomes, the symptoms, time points, formula, and dosage of Chinese medicine should be recorded in detail. More researchers had focused on this and built a tailed reporting guideline for TCM, such as the extension of PRISMA for acupuncture (Liu et al., 2014; Li et al., 2017). Meanwhile, it is also essential to create a series of standards and guidelines for TCM to improve its evidence quality in clinical practices and research.

As mentioned previously, treatments from TCM are dialectical and emphasize individual therapy based on the time, place, and patient. When designing a clinical study, it is difficult to conduct large-sample, multi-center, double-blind RCTs due to differences in the treatment form, method, and nature (duration and intensity), and may introduce higher bias and risk (Xie and Leung, 2009). However, by ignoring the diversities of TCM in a systematic review, the evidence cannot be translated into practice effectively. Thus, researchers are suggested to minimize the random error using rigorous, transparent, and standardized design and to also reduce the system error using statistical methods when conducting small sample clinical trials and are advised to report both quantitative and qualitative results of primary studies and systematic reviews.

In addition, we found that the reviews published in Chinese rarely provided information about their protocol. A published or documented detailed plan could promote consistency between the authors’ plan and action when implementing the research, ensure clear responsibility, and enhance the openness, transparency, and repeatability of evidence (Liu et al., 2021). It also helps to reduce the authors’ bias when selecting and extracting (Moher et al., 2009). However, there is no registration platform for TCM yet, which will also be an important issue in the future.

## 5 Conclusion

The reporting quality in the abstracts of systematic reviews/meta-analyses about TCM for ischemic stroke is poor and does not facilitate rapid access to valid information for clinical practitioners. The methodological quality is of a medium level, but this evidence lacks certainty, especially with a high risk of bias in individual studies.

In addition, rare reviews reported the process of protocol registration and the list of excluded studies, which increases the repetitive crisis. Although TCM has its advantages for ischemic stroke, it is necessary to develop target guidelines to improve its evidence quality.

## Data Availability

The original contributions presented in the study are included in the article/Supplementary Material; further inquiries can be directed to the corresponding author.
